# Measurement of harms in community care: a qualitative study of use of the NHS Safety Thermometer

**DOI:** 10.1136/bmjqs-2017-006970

**Published:** 2017-12-02

**Authors:** Liz Brewster, Carolyn Tarrant, Janet Willars, Natalie Armstrong

**Affiliations:** 1 Lancaster Medical School, Lancaster University, Lancaster, UK; 2 Department of Health Sciences, University of Leicester, Leicester, UK

**Keywords:** nurses, quality improvement, quality measurement, qualitative research

## Abstract

**Objectives:**

Measurement is a vital part of improvement work. While it is known that the context of improvement work influences its success, less is known about how context affects measurement of underlying harms. We sought to explore the use of a harm measurement tool, the NHS Safety Thermometer (NHS-ST), designed for use across diverse healthcare settings in the particular context of community care.

**Methods:**

This is a qualitative study of 19 National Health Service (NHS) organisations, 7 of which had community service provision. We conducted ethnographic observations of practice and interviews with front-line nursing and senior staff. Analysis was based on the constant comparison method.

**Results:**

Measurement in community settings presents distinct challenges, calling into question the extent to which measures can be easily transferred. The NHS-ST was seen as more appropriate for acute care, not least because community nurses did not have the same access to information. Data collection requirements were in tension with maintaining a relationship of trust with patients. The aim to collect data across care settings acted to undermine perceptions of the representativeness of community data. Although the tool was designed to measure preventable harms, care providers questioned their preventability within a community setting. Different harms were seen as priorities for measurement and improvement within community settings.

**Conclusions:**

Measurement tools are experienced by healthcare staff as socially situated. In the community setting, there are distinct challenges to improving care quality not experienced in the acute sector. Strategies to measure harms, and use of any resulting data for improvement work, need to be cognisant of the complexity of an environment where healthcare staff often have little opportunity to monitor and influence patients.

## Introduction

Improving the quality and safety of healthcare is a system-wide priority, and the role of measurement to assess care quality and to inform improvement is widely recognised as integral.^1^ Improvement programmes often use measurement to assess the quality of care, and the resulting data need to be reliable and accurate. Lack of robust data and problems with measuring care quality have been repeatedly discussed in the literature.^2 3^ Poor-quality data mean it may be difficult to convince sceptics that there is a problem, or to evaluate the effectiveness of improvement initiatives.^4 5^ The process and practicalities of collecting these data are often glossed over in the literature, but measurement is integral both to surfacing problems and to evaluating the success of any intervention to address these.^6 7^

Measuring care quality and making improvements is not a straightforward process.^8^ Evidence shows that context affects the success of improvement initiatives, and that what works in one location may not work in another.^9^ Replicability and transferability have affected the implementation of measurement tools within the acute setting. Differences between the acute, primary and community care settings may further complicate attempts to transfer measures.^10–12^ Here we focus on the use in community care of a harm measurement tool designed to be used across care settings. Community or district nursing has a central role in care provision in the UK.^13^ It supports patients in their homes or residential care homes by providing complex care outside the hospital. Patients are often older adults or have long-term health conditions that require monitoring to avoid exacerbation; other duties include palliative care, drug administration and wound care. Referral to community nursing can facilitate earlier discharge from hospital. The patient’s home is positioned as a key location for care delivery, but it is a complex and non-standard setting that may affect providers’ ability to deliver high-quality care.^14 15^

The NHS Safety Thermometer (NHS-ST) was designed to facilitate the measurement of harms across different healthcare contexts, providing data that could be used to inform local improvement work. The NHS-ST was intended to generate data that could be used to support local improvement, but it does not directly link to any particular improvement programme. Developed in 2010, it is a point-of-care instrument that collects prevalence data on four common harms (pressure ulcers, falls, urinary tract infections (UTIs) (in patients with catheters) and venous thromboembolism (VTE)) (table 1).^16 17^ These harms were considered as preventable by improvements in care delivery. Using a standardised instrument, nursing staff collect data regardless of the location of the patient (eg, home, nursing home, hospital) to enable a ‘temperature check’ of harm across the National Health Service (NHS).

**Table 1:** Guidance and definitions for use of the NHS Safety Thermometer

Age	
Collected in three age bands	Values: <18, 18–70, >70
Gender	Values: male, female
Old pressure ulcers	
Old pressure ulcers developed within 72 hours (3 days) of admission to organisation. The category of the patient’s worst old pressure ulcers is recorded.	Values: none, cat 2, cat 3, cat 4
New pressure ulcers	
New pressure ulcer developed 72 hours (3 days) or more after admission to organisation. The category of the patient’s worst new pressure ulcers is recorded.	Values: none, cat 2, cat 3, cat 4
Patient falls	
Any fall that the patient has experienced within the previous 72 hours in a care setting (including home if the patient is on a district nursing case load) The severity of the fall is defined in accordance with the National Reporting and Learning System categories.	Values: none, no harm, low harm, moderate harm, severe harm, death
Catheters	
An indwelling urethral urinary catheter in place at any point in the last 72 hours Record the number of days that it has been in place. If the patient has not had indwelling urethral urinary catheter in place at any point in the last 72 hours, record no catheter.	Values: 1–28 days, 28+ days, days unknown, no catheter
UTIs (urinary tract infections)	
Any patient being treated for a UTI Record if the treatment started before the patient was admitted to your organisation (old) or after admission to your organisation (new). Treatment for a UTI is based on clinical notes, clinical judgement and patient feedback.	Values: no UTI, old UTI, new UTI
VTE (venous thromboembolism) assessments	
Is there a documented VTE risk assessment?	Values: no, yes, N/A
VTE prophylaxis	
If the patient is at risk, has VTE prophylaxis started?	Values: no, yes, N/A
VTE treatment	
If the patient is being treated for VTE, choose the type of VTE. Use old VTE where the patient had the VTE before admission. Use new VTE where the patient developed the VTE after admission.	Values: no VTE, old DVT (deep vein thrombosis), old PE (pulmonary embolism), old other, new DVT, new PE, new other

N/A, not applicable.

Introduced in response to the NHS Quality, Innovation, Productivity and Prevention programme’s identification of the need for a robust data set, which looked across the healthcare system to measure avoidable harms and drive improvement,^18^ the NHS-ST represents the first attempt by the English NHS to measure harms at scale across diverse health settings. Since 2012, the Commissioning for Quality and Innovation (CQUIN) scheme has incentivised NHS care providers to measure these four harms using the NHS-ST on all patients (with some exceptions) on a predetermined date each month.^19^ Across participating organisations, approximately 200 000 patients per month are screened for harm.^20^

Drawing on a broader study evaluating the NHS-ST’s underlying principles and use in practice, this paper focuses on its use in community care. A perceived strength of the NHS-ST is its applicability across all care settings^18^; we sought to explore how the tool was perceived and used in community care.

## Methods

Our ethnographic study drew on observation, interview and documentary data. We collected data in 19 NHS organisations, 7 of which had community service provision. Organisations were sampled purposively to reflect their size and type, and reported levels of harm on the NHS-ST. We conducted ~115 hours of observation across organisations (~49 hours in the community setting), with the aim of understanding the tool in use and to map the process of data collection.

To support the observations, we conducted interviews with front-line staff (52; 18 in the community) and senior staff (38; 13 in the community). The broader evaluation was informed by interviews with senior national NHS leaders (4), identified experts with specialist knowledge of the four harms (27), and those involved in developing and/or implementing the NHS-ST (5). Observation fieldnotes were debriefed, audio-recorded and transcribed. All 126 interviews were audio-recorded and transcribed verbatim. Relevant documents were collected in each organisation.

Data analysis was based on the constant comparative method.^21^ Through comparison across transcripts, documents and fieldnotes, initial open codes were organised into thematic categories, which provided a framework for processing all data using QSR NVivo software.^22^ The data presented in this paper are mainly drawn from observations in which we shadowed community nurses in their daily duties, and interviews with front-line and senior staff conducted in seven community organisations. Our approach to analysis looked across the whole body of data at the differences in how the NHS-ST was used in diverse care settings. In analysis of all data collected, the differences between community and acute settings came into sharp focus and showed the challenges of designing a tool that takes a whole system approach.

The evaluation was designated as service evaluation under the NHS Research Ethics Framework and was registered as such with each participating organisation.^23^

## Findings

We identified distinct challenges in the community setting that were not found in the acute setting, and concerns about the extent to which measures can be easily transferred across care settings. First, priorities for the harms to be measured in community care were often different from other settings. Second, the value of measurement was often not realised in the community setting, largely because of doubts about the extent to which the harms being recorded were preventable. Third, there were challenges in actually collecting the data. Although the NHS-ST was designed with input from both community and acute nursing teams,^24^ facilitating measurement in the community context was still a demanding process. Staff saw recording accurate information about harms as problematic because they frequently did not have access to relevant information sources. The data collected were not seen as credible, because staff felt they did not present an accurate picture of harms in the community.

### What to measure: relevant harms in community care

Community staff believed that different harms were priorities for measuring and addressing within community settings as compared with acute settings. There was criticism of the harms that had been included in the NHS-ST and their applicability to the community setting. A key issue was the inclusion of VTE risk assessment. Despite instructions that data should not be recorded in the community setting, this still took time to complete through constant selection of ‘Not Applicable’ and reinforced the view that the tool had been designed primarily for the acute sector.

I think it’s a shame, again the Safety Thermometer was probably set up for the acute model because of course none of the community teams do VTE risk assessments. That is still an issue for us. Because the VTE element of the Safety Thermometer has to be clicked for each individual line and of course it’s not applicable for community teams. And if you’ve got 50 patients you’ve got to do and it’s not applicable that has to be changed. (Site B, Senior Staff 1)

Community staff suggested numerous other harms that they felt were priorities in their setting that were not measured by the NHS-ST. Nutrition, hydration, medication errors, fractures, safeguarding and care transfers were all harms that were mentioned as more relevant to the community sector than VTE. There appeared to be some appetite for different versions of the NHS-ST in different care settings, rather than one uniform tool used across the health sector. Staff argued for the need to tailor the tool to ensure that its content was aligned with the context in which it was being used.

Safety Thermometer is a one size fits all and let’s shoehorn your services into it, as opposed to making it relevant to those areas.…[Something that] is a really big harm to patients [in the community] – everyone is talking about integration of services – but we’ve still got poor transfers of care happening, so that’s a fantastic measure to look at because that really harms the patient. (Site A, Senior Staff 2)

### The purpose of measurement: harm prevention in community care

Community providers questioned the purpose of harm measurement through the NHS-ST. Staff often reported little use of the data that they collected and questioned what they would meaningfully gain from doing so. There was some evidence from more senior staff that the data did feed into wider improvement strategies and were used to help to drive up quality of care, but on the whole, collecting the data was not seen by front-line staff as being connected with improving patient outcomes.

There’s nothing specific that I’m aware of that we get fed back that makes any impact on our workloads. (Site Q, Front-line Staff 1)

Because front-line staff on the whole believed the data they collected had little relevance for their practice in the community, or for improving their practice at point of care, many reported little interest or engagement with the NHS-ST. They could not see the benefit in data collection—they just saw the data as ‘feeding the data beast’ or ‘going into a black hole’—it was not relevant for them. Overall, the lack of alignment of the methods and content to the specific context of community care undermined its value as a tool to drive improvement.

A core principle of the NHS-ST was that the harms measured were predominantly preventable ones that could be reduced or avoided through improvements in care. While the preventability of the chosen harms in the acute setting was subject to some debate, in the community setting the presumption of preventability of these harms was highly contested. Community staff suggested that the harms measured by the NHS-ST were often outside their control.

A key issue was the perception that influence over patient behaviour and actions in the community was often limited. Patient autonomy was greater in the home setting, and community nurses were realistic about their ability to compel a patient to adhere to clinical advice when they were not present to encourage compliance.

I can’t do anything about that patient’s house [to prevent falls]. I can’t make her take all her rugs up. I can’t make her use her zimmer frame properly when I’m not there. (Site A, Senior Staff 2)

And if [harms like pressure ulcers] do occur within our care it is because of underlying health that’s causing them to have non-compliance, non-concordance. Or the very terminally ill – the family, it’s always the family: ‘please don’t move them, please don’t touch them’. (Site C, Front-line Staff 2)

Although nurses in hospitals also contended with issues of adherence to their clinical advice, their ability to monitor patients and control the environment was greater. Community nurses could ensure patients were provided with equipment to reduce their risk of harm, such as pressure ulcer cushions and walking frames, but they had little influence over whether patients actually used the equipment provided, or even that they would agree to have it at all. It was often difficult to get patients to agree to changes to their environment that would improve safety.

Front-line staff therefore felt that harms recorded in this environment did not necessarily reflect poor care on their part—they could not be responsible for what patients did (or did not do) when they were not there.

[The nurse] asked [the patient] if he had any sores and he said no. She said – have you been using the [pressure ulcer prevention] cushion I got for you? He said he’d tried it for a couple of days but it had made him sore, so now he uses it to prop up his legs when he’s sitting down (as they get swollen). (Site O, observation debrief)

In the community setting, ensuring that pressure ulcers were treated at an appropriately early stage was particularly challenging as it required patient disclosure or permission for a physical examination. Along with this, community staff felt that advice given by the NHS-ST team about having a regular turning or repositioning schedule to prevent pressure ulcers was difficult to apply in the community setting in which a nurse was not physically present throughout the day and night.

[The community nurses] go out and they see the patient and they see the foot and the pressure ulcer is healed: ‘fantastic, is everything else OK?’ And the carer for the person says: ‘yes, everything is fine’. But a week later the carer phones up and says: ‘oh they’ve got a sore on their bottom’. And it turns out that that is a grade 3 pressure ulcer. (Site C, observation debrief)

A lot of the [guidance about how to prevent harm] that had come through needed almost tailoring to fit community nursing. That whilst some of it was extremely relevant in a hospital, where you do have that 24/7 availability to care for the patient, that that wasn’t applicable in the patient’s home. (Site A, Senior Staff 2)

### Data collection in community care

Data collection for the NHS-ST was often not straightforward in community care. Community nurses did not have the same access to information as data collectors within acute settings. In the hospital setting, nursing care is provided 24 hours a day, 7 days a week, although patients are not constantly supervised. In the community setting, patients are in their home and nurses visit at agreed times. Whether and when a nurse was present affected what data they could collect.

One significant difference between acute and community settings was the extent of access to tests and test results to confirm diagnoses. For example, when catheterised patients in hospital settings were suspected of having UTIs, diagnostic tests were commonly carried out and their results were available on review of patient notes. In contrast, community nurses did not have easy access to facilities to test whether an infection was present, or might not have knowledge of an infection if it had been diagnosed and treated by a general practitioner.

[UTIs in a catheterised patient], that’s very different from the hospital. Because when they’re in hospital they’re in bed, they’re on an antibiotics course. Sometimes our patients are on long-term catheters, we don’t see them for three months. They may have a UTI, they will phone the doctors themselves, they will input a specimen themselves. We won’t get to hear about it. (Site C, Front-line Staff 1)

In hospital settings, nurses were usually able to review complete sets of patient notes and conduct physical examinations of patients as part of their data collection if required. In a patient’s home, nurses had to rely more on verbal report, with the patient or their carer alerting them to a potential harm such as a UTI or pressure ulcer. Data collection involved asking the patients additional questions, for example: Do you have any sore areas? Have you had a fall recently? This was seen as less reliable than data collected within hospitals, which were based on more objective information. In particular, there was the risk that patients or carers would fail to report harms to the community nurse.

We’ve got patients that will say ‘I had another fall but please don’t tell Dr [name], please don’t – I don’t want anything [equipment]’. I bet out of ten I bet only one or two would let us refer them totally for the falls [assessment]. (Site K, Front-line Staff 1)

Data collection requirements could be in tension with the need to maintain a relationship of trust with patients and to respect patients’ autonomy and dignity in their homes. Collecting reliable data for the NHS-ST could require a change in the community nurses’ approach to working with patients. For example, nurses were now requesting physical examinations with patients. Community nurses described problems with this shift, including disruption to their long-standing relationships with patients.

[The nurses] had known these people for a long time and they were saying things like: ‘I have gone and seen this patient once a week for the past year, and now suddenly I am asking every time I go round: ‘can I look at your bottom? Can I check for pressure ulcers?’’ And patients are suspicious of this and they are not really on-board with the idea…They are not necessarily keen on the idea of letting the nurse into their personal space. (Site K, observation debrief)

Community nurses needed to maintain a long-term relationship with the patient in order to deliver care, and believed they could not push things too far as patients at home had greater scope for refusing to comply with examination and treatment than in the hospital setting. One nurse was observed using her NHS-ST form to record that the patient refused to be physically examined, as she felt unable to record any other information and could not state with any certainty whether harms had occurred. Another nurse cited an example of a patient who would only allow her, but not any other nurse, to examine his pressure ulcer (in an intimate area). Thus, the availability of accurate data could be affected by the nurse on duty on data collection day. The fact that community nurses were not, as part of their role, routinely giving patients full physical examinations meant that harms were not always identified during visits. In the acute setting, there were further opportunities for the collection of information when, for example, washing or dressing patients.

A further issue was that data collection schedules that were designed to enable representative data to be collected across the health economy were, conversely, seen as potentially undermining the representativeness of community data. To ensure consistency and coordination, data collection days were set to fall on the first or second Wednesday every month, meaning that all organisations collected their data on the same date. The choice of Wednesday was not problematic for data collection in acute settings as, typically, the case mix of patients in hospitals on a Wednesday did not differ systematically from that on other days.

In community settings, however, the data collection day was consequential for the sample of patients included. Because community nurses do not see every patient on their case load every day, they only recorded patients who they visited that day. Community staff found this challenging, because they knew that patients with different needs were seen on different days. For example, patients who were more unwell would need to be seen daily or three times a week (typically Monday, Wednesday and Friday), whereas patients who were less unwell would be seen twice-weekly. Community nurses felt that the majority of their overall case load was these twice-weekly, less unwell patients, but that the patients seen on a Wednesday, and therefore captured in the NHS-ST data collection, were more likely to be the acutely ill patients, which might skew the data and lead to disproportionate numbers of harms being recorded.

If patients are seen twice weekly it’s normally Monday/Thursday or Tuesday/Friday, because Wednesday is a very obscure day really. And [patients seen on Wednesday] would only be seen the three days a week or daily. If you were actually looking at the whole caseload it may give you more validated data really, because all you are asking for is patients seen today. Well, it’s not telling you anything. (Site C, Front-line Staff 3)

The day of the week that data were collected had an impact on front-line and senior staff’s view of the data’s credibility and representativeness.

In addition, several participants mentioned that they felt the NHS-ST was open to ‘gaming’ or the potential for purposefully skewing figures to meet CQUIN or local improvement targets, particularly if organisations were concerned about trying to avoid oversampling acutely ill patients. This possibility undermined trust in the data.

Whilst you’re in a hospital setting, you’re seeing every single patient on that setting on that day and that’s fine. There are so many different varying factors that can determine which patients you see on that particular day in the community and if we wanted to be a really, really target driven organisation without any care for our patients we could say to all our staff: ‘right, we’ll hit our targets – you don’t go and see any patients with a pressure ulcer on a Wednesday’. (Site K, community organisation, Senior Staff 1)

The downside of the Safety Thermometer is you’re relying on professional and clinical integrity. Because, actually, it would be in the clinician’s interest – they know we collect the data on a Wednesday – to see all the patients that they know have no harms. So when we produce the report, they’re [a] hundred percent harm-free. And I think there could be an element of that going on with the Safety Thermometer throughout the country. (Site O, Senior Staff 1)

Another feature of the community setting that made the collection of robust data particularly challenging was the fact that community nurses conducted the data collection alone, as part of their routine patient visits. Acute sector nurses often commented that they had conversations with colleagues while collecting data in an effort to ensure that harm definitions were applied consistently. Colocation of staff in the acute setting gave opportunities for discussion with the patient present, which was particularly relevant when trying to categorise pressure ulcers correctly. In contrast, community nurses did not have these opportunities for checking and validating their decisions about data recording. The tool asked staff to record if a harm was ‘old’ or ‘new’, with the differentiation being whether treatment began before or after admission. Although this could be interpreted as ‘admission to the caseload’ of a community nurse, this was not always applied.

[District nurse] was talking to me in the car and was saying that actually the terminology, the wording on the Safety Thermometer is difficult to understand. She doesn’t understand the difference between an old pressure ulcer and a new pressure ulcer really because they’re all old to her, and so it’s not a distinction that’s important. And actually what is important to her is knowing where it was acquired, was it acquired in the hospital or the patient’s home or what? So she put all of them down as old. (Site G, observation debrief)

These challenges in when and how to collect data impacted on front-line and senior staff’s use of the NHS-ST tool and their view of the data it produced.

## Conclusion

While the NHS-ST as a measurement tool was designed to be transferable across care contexts, in practice it was understood and experienced by healthcare staff as socially situated. Principles intended to make the NHS-ST easy to use across all care settings contributed paradoxically and raised concerns about the relevance of the tool in the community setting. The context in which staff were working and collecting the data raised questions about the validity and representativeness of the data produced. Many questioned to what extent the tool, and the data derived from it, could meaningfully be used for any subsequent improvement activity because of ambiguities around where and with whom responsibility for any harm lay, and to what extent any harm was ultimately preventable. These concerns about the tool and the data collected through it were likely to influence any subsequent improvement work based on them.^8^ If the data were not considered relevant or accurate, then they were less likely to have credibility and be taken forward into local improvement initiatives.

Designing a measurement tool that can be used across a whole healthcare system is challenging because of contextual features of the care settings in which it will be used. While acute settings were well-bounded, with nurses acting within defined ward settings, the community setting was often complex. Care delivered in the community is organised differently, with patients seen more or less frequently depending on clinical need. This undermined attempts to collect prevalence data that were regarded as meaningful. Monitoring and measurement in a community or home setting are perceived as more difficult, and availability of data on quality and safety is generally regarded as in need of improvement.^25^

Community nurses were not always able to mandate action to prevent harms, and this meant that they felt the emphasis on prevention of the harms they were recording was misplaced. Previous research has identified the boundaries of the community nursing role as needing careful negotiation between healthcare professional and patient to facilitate treatment.^26 27^ Here, nurses queried to what extent improvements could be made in their context. This undermined acceptance of the measurement activity and the ability of the resulting data to contribute helpfully to improvement.

By examining the interaction between the intervention (the NHS-ST) and the context (the community setting), we have highlighted that the social and cultural conditions within which a tool is used affect its ability to perform as designed.^28^ Although the NHS-ST was designed to work across the whole health system, in practice this involved several compromises. These compromises were felt by staff across acute and community settings, showing that designing a tool that provides meaningful data in all healthcare settings is a challenge. However, this has been recognised within the ‘next generation’ NHS-ST tools, several of which have a community and acute version with different definitions.

This paper has shown that when care is delivered beyond hospital boundaries, there needs to be attention paid to the context in which measurement takes place. It provides greater understanding of the under-researched context of measurement in the community setting.^29^ The community setting presents distinct challenges to collecting data to encourage improvements in care quality, although they are not insurmountable. Context is known to explain why the replication of previously successful improvement interventions is problematic.^28^ Strategies to collect data to use in improvement need to take into account the particular difficulties of collecting data and implementing innovations in an environment where healthcare staff have fewer opportunities to monitor and influence patient action. The socially situated nature of measurement needs to be taken into account. Previous work examining measurement has identified that one unintended consequence of trying to measure things is insensitivity, with the tool as a ‘blunt instrument’ that does not take into account the nuances of everyday care work.^30^

As models of healthcare delivery change, bringing together acute and community service provision under umbrella organisations such as the new models of care (Vanguards) in the UK,^31^ and accountable care organisations in the UK and more widely, the need to work across community and acute settings will continue to grow. The challenge of providing safe and continually improving care will be experienced at greater scale and the knowledge gathered from the implementation of the NHS-ST may help to inform future good practice.
